# Supplementary material for: Pyruvate Kinase M2 Promotes Hair Regeneration by Connecting Metabolic and Wnt/β-Catenin Signaling
Source: Pharmaceutics. 2022 Dec 13;14(12):2774. doi: 10.3390/pharmaceutics14122774 (PMC9781674; doi:10.3390/pharmaceutics14122774)
Supplement: Supplementary file 1 [file pharmaceutics-14-02774-s001.zip › Revision Supplemental material_Pharmaceutics_PKM2-hair.docx]

**Supplementary material**

**Supplementary figure 1. Effects of the control of PKM2 activity on hair elongation.** (A) The relative expression level of PKM2 mRNA in the scalp skin (GEO: GSE45512, n=5). (B-C) The vibrissa follicles of 6-week-old C57BL/6N female mouse were isolated and treated with DMSO or TEPP-46 as described in Materials and Methods. (B) Images of mouse vibrissa follicles and (C) quantitation of the length of vibrissa follicles at day 10 (n=13). (D-E) The mouse dorsal skins were shaved and topically applied daily with vehicles, TEPP-46 or MNX (150 mM) for 14 or 35 days. (D) Quantitative measurements of hair weight for dorsal skins treated with TEPP-46 or MNX for 35 days (n=5). (E) Quantitative estimation of PCNA-positive cells of hair follicles treated for 14 days around the stem cell region (n=5). (F-G) Mouse vibrissa follicles were separated and incubated with DMSO or shikonin. (F) Gross images of vibrissa follicles and (G) quantitation of vibrissa follicle length at day 10 (n=10). (H) The mouse dorsal skins were plucked and topically treated with vehicle or shikonin (0.1 mM) daily for 5 days. Quantitative evaluation of PCNA-positive cells around the stem cell region (n=3). Scale bars = 200 µm. Values are expressed as mean ± SEM. Student’s t-test (* P < 0.05, ** P < 0.005, *** P < 0.0005).

**Supplementary figure 2. Effects of Wnt/β-catenin signaling activator on PKM2 level.** (A-C) The dorsal skins of 8-week-old C57BL/6N male mice were plucked and harvested on the indicated days. (A) IHC staining for PKM2, β-catenin and PCNA for dorsal skins. Magnified images indicate the stem cell region. (B) Quantitation of the intensity of the fluorescence signals for nuclear β-catenin and PKM2 around the stem cell region (n=15). (C) Quantitation of PCNA-positive cells around the stem cell region. (D) Mouse dorsal skins were shaved and applied daily with vehicles, KY19382 (0.5 mM), or MNX (100 mM) for 14 days. IHC staining for PKM2, β-catenin and Keratin 15 in the hair follicles of skins. Magnified images indicate a stem cell region. Scale bars = 100 µm. Values are expressed as mean ± SEM. Student’s t-test (* P < 0.05, ** P < 0.005, *** P < 0.0005).

**Supplementary figure 3. Influences of combined treatment with TEPP-46 and KY19382 on proliferation.** (A) The dorsal skins of 7-week-old C57BL/6N male mice were shaved and topically applied with vehicles, KY19382 (0.5 mM), TEPP-46 (0.5 mM), or MNX (150 mM) daily for 14 days. Quantitative calculation of the number of PCNA-positive cells (n=4). (B) Mice wounds were treated daily with each compound for 14 days. Quantitative measurements of PCNA-positive cells (n=5). Values are expressed as mean ± SEM. Student’s t-test (* P < 0.05, ** P < 0.005, *** P < 0.0005).

**Supplementary figure 4. Expression profiles of PKM2 during the wound-induced hair neogenesis.** The dorsal skins of 3-week-old C57BL/6N male mice were wounded and sacrificed on the indicated days after wounding. (A) H&E staining of wounded skins. (B) IHC staining for β-catenin, PKM2, Keratin 17, and Fgf 9. Magnified images indicate the neogenic follicles. Relative *Pkm2* mRNA level (C) and the evaluation of PK activity (D) of wounded skins (n=5). Scale bars = 100 µm. Values are expressed as mean ± SEM. Student’s t-test (* P < 0.05, ** P < 0.005, *** P < 0.0005).
