# Supplementary figures and images for: Pyruvate Kinase M2 Promotes Hair Regeneration by Connecting Metabolic and Wnt/β-Catenin Signaling
Source: Pharmaceutics. 2022 Dec 13;14(12):2774. doi: 10.3390/pharmaceutics14122774 (PMC9781674; doi:10.3390/pharmaceutics14122774)

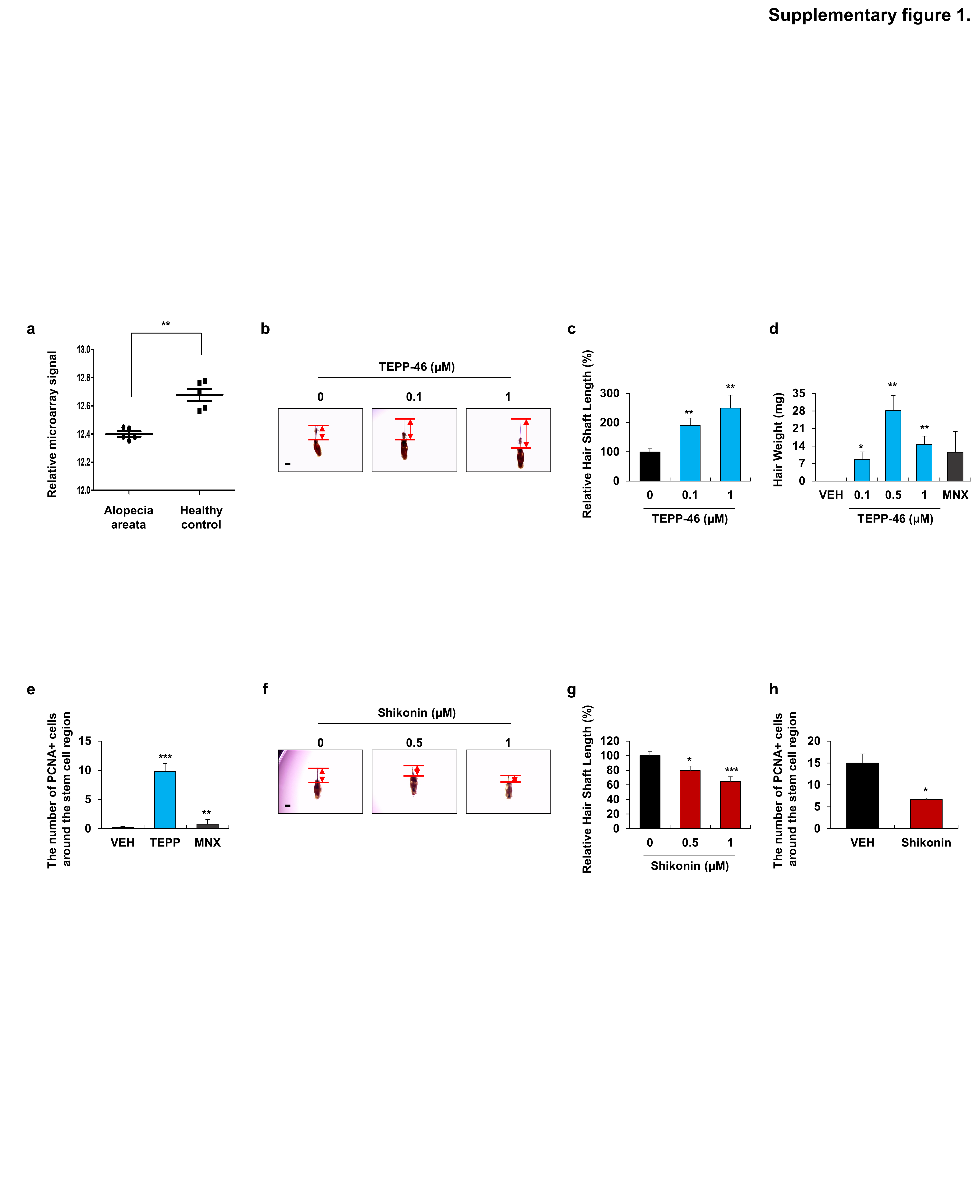

Supplement: Supplementary file 1 [file pharmaceutics-14-02774-s001.zip › Supplementary figure 1.TIF]

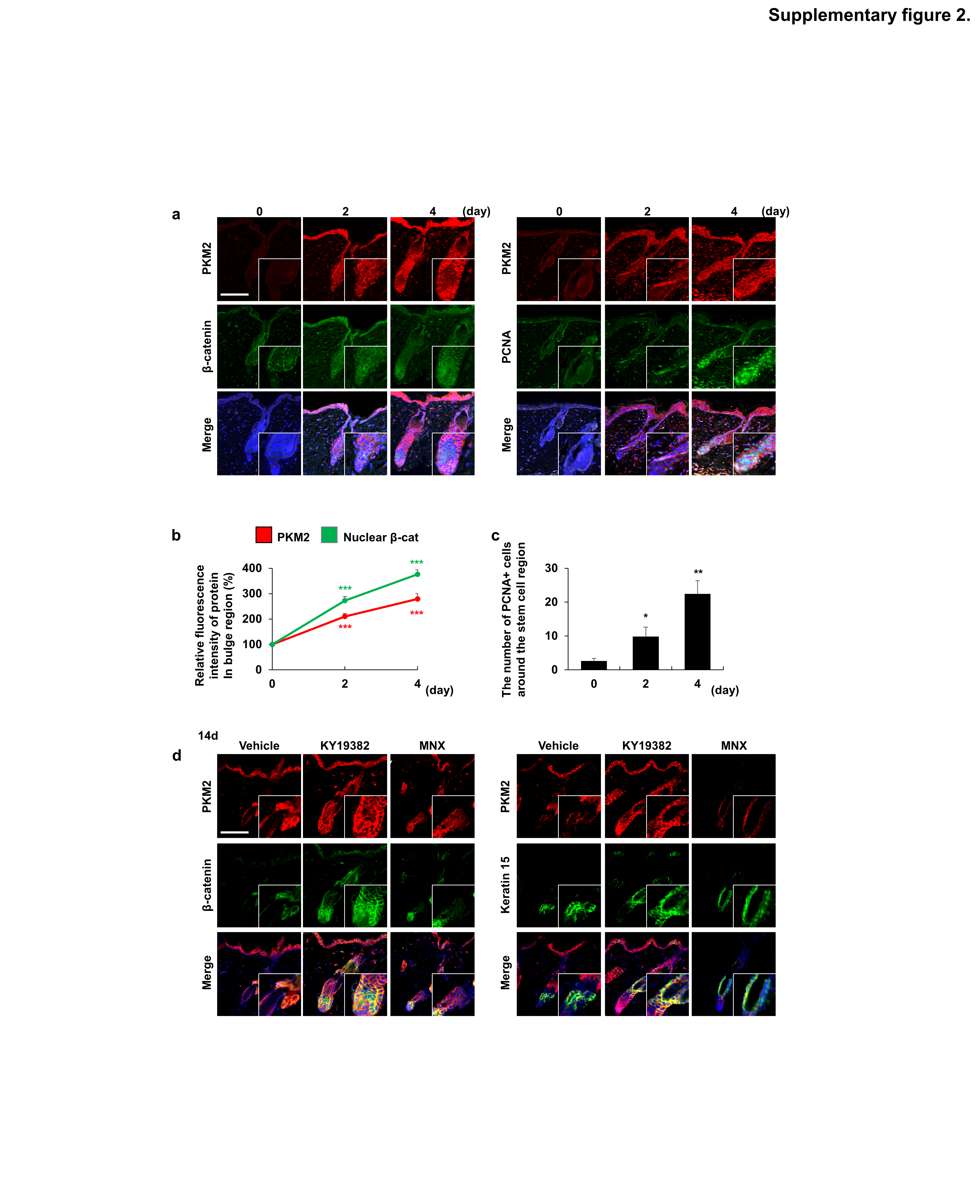

Supplement: Supplementary file 1 [file pharmaceutics-14-02774-s001.zip › Supplementary figure 2.TIF]

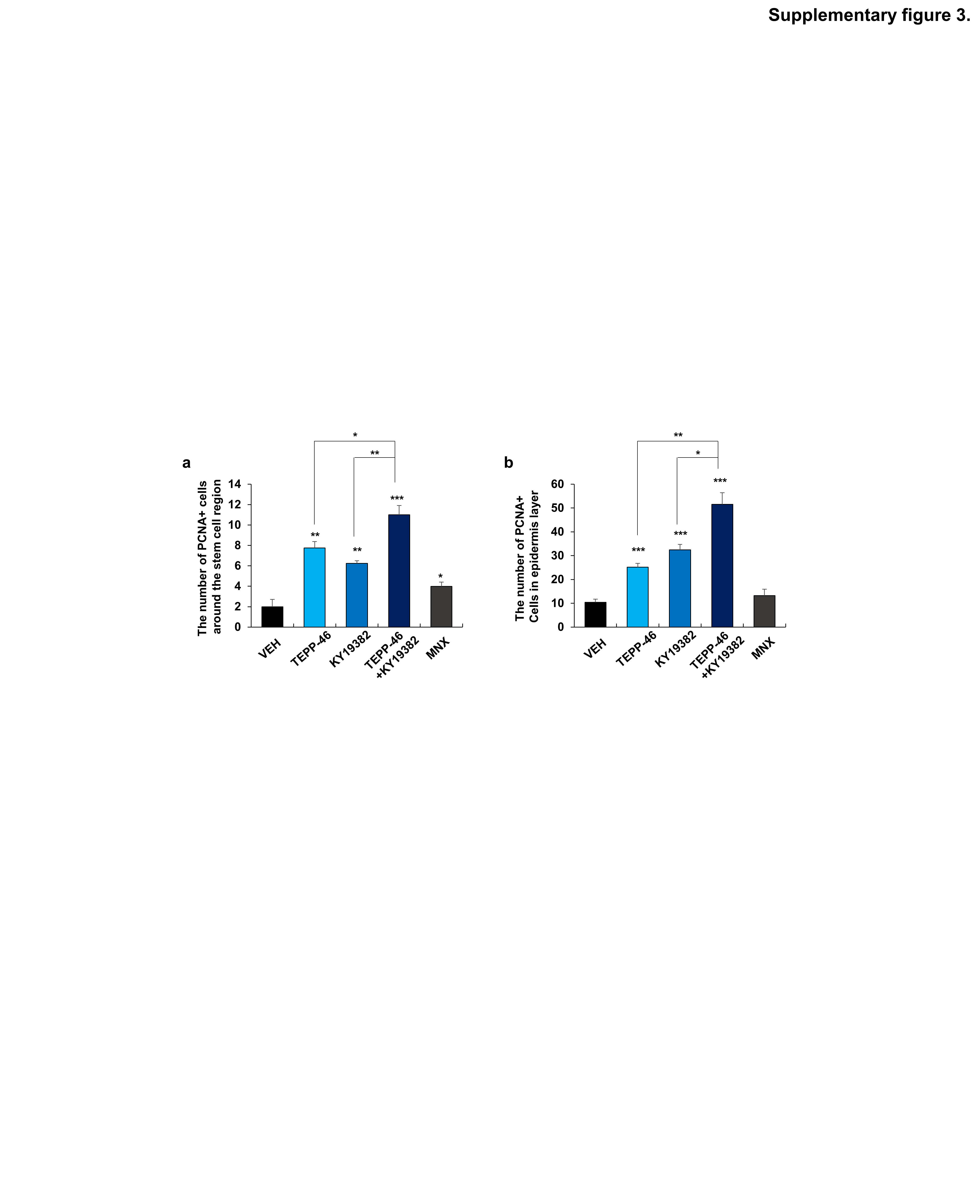

Supplement: Supplementary file 1 [file pharmaceutics-14-02774-s001.zip › Supplementary figure 3.TIF]

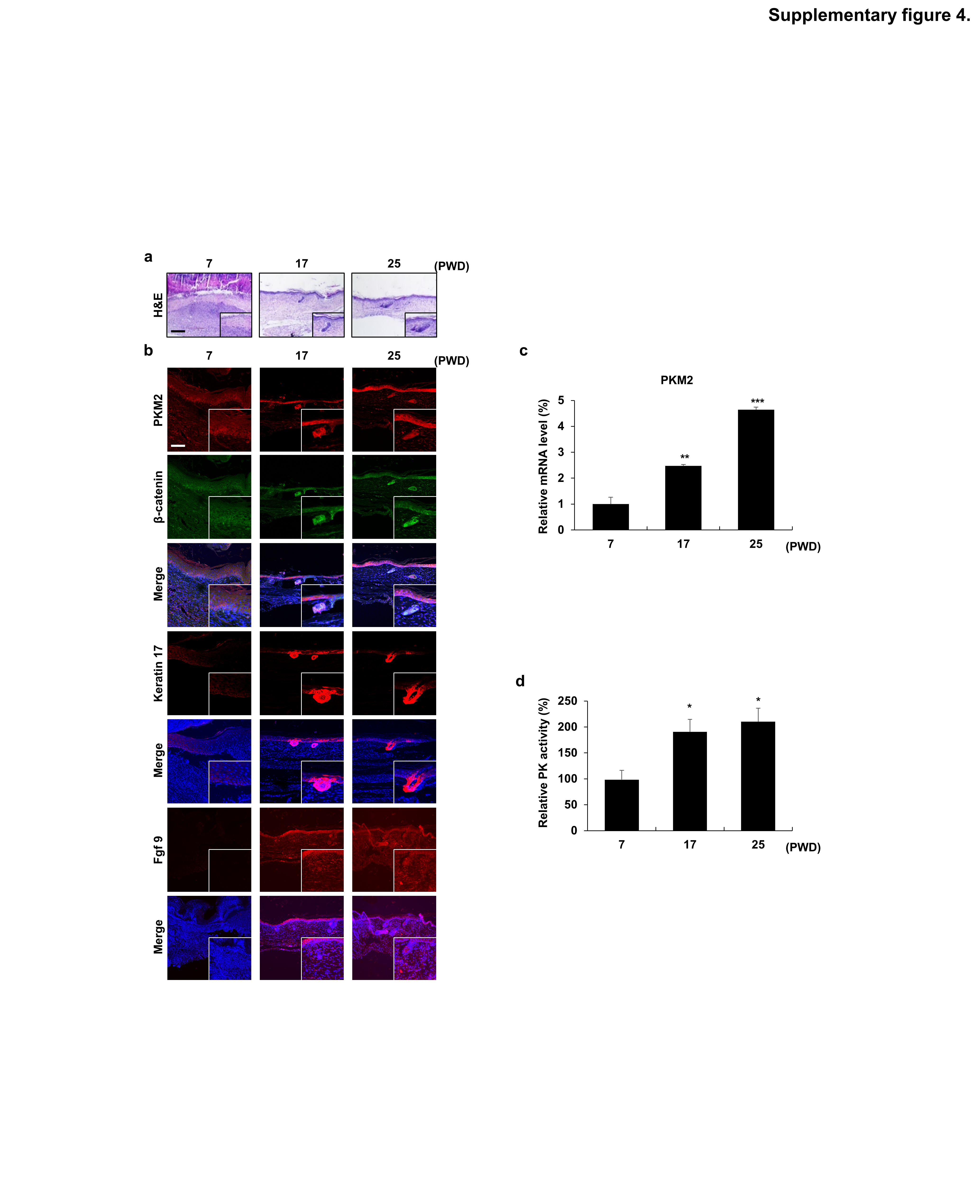

Supplement: Supplementary file 1 [file pharmaceutics-14-02774-s001.zip › Supplementary figure 4.TIF]
